# CD97 serves as a novel biomarker of immune cell infiltration in hepatocellular carcinoma

**DOI:** 10.1186/s12957-022-02829-2

**Published:** 2022-12-04

**Authors:** Qiuming Su, Lu Li, Xiaokai Li, Wang Li, Xibing Zhang, Yun Dong, Lei Han, Duo Wang, Jianghua Ran

**Affiliations:** 1 Department of Hepatopancreatobiliary Surgery, The Affiliated Calmette Hospital of Kunming Medical University, 1228 Beijing Road, Panlong District, Kunming City, 650224 Yunnan Province China; 2 Department of Hepatobiliary Surgery, The First Affiliated Hospital of Kunming Medical University, Kunming City, Yunnan Province China

**Keywords:** CD97, Hepatocellular carcinoma, Prognosis, Immune infiltration, Tumor-associated macrophages

## Abstract

**Background:**

CD97 is the most widely expressed G protein-coupled receptor in the epidermal growth factor seven-span transmembrane family. It plays a vital role in cell adhesion, migration, and cell connection regulation. We explored the role of CD97 in hepatocellular carcinoma (HCC).

**Methods:**

We evaluated CD97 mRNA expression in HCC using TNMplot and the Gene Expression Omnibus database. The clinical prognostic significance of CD97 in HCC patients was evaluated by gene expression profiling interactive analysis, the Kaplan–Meier plotter, and the UALCAN database. The Tumor Immune Estimation Resource (TIMER) and CIBERSORT databases were used to analyze the relationships among CD97, genes positively related with CD97, and tumor-infiltrating immune cells.

**Results:**

CD97 was highly expressed in HCC tissues and was associated with an adverse prognosis. CD97 and genes positively related with CD97 were positively correlated with the abundance of tumor-infiltrating immune cells and strongly correlated with tumor-infiltrating macrophages (all *r* ≥ 0.513, *P* < 0.001). CD97 was positively correlated with M2 macrophage and tumor-associated macrophage markers (both *r* ≥ 0.464, *P* < 0.001). CD97 was found to be an immune-related gene in HCC and positively correlated with the TOX, PD-L1, PD-L2, CTLA4, and PD-1 immune checkpoint genes. CD97 copy number alterations affect the level of immune cell infiltration and mRNA expression.

**Conclusions:**

CD97 can be used as a potential molecular marker of prognosis in HCC, which is associated with immune cell infiltration.

**Supplementary Information:**

The online version contains supplementary material available at 10.1186/s12957-022-02829-2.

## Background

Hepatocellular carcinoma (HCC) is one of the most common malignant tumors worldwide. The incidence and mortality rate of HCC rank sixth and third, respectively [1], posing a severe threat to human health. HCC is increasing in incidence in China and is the primary causative factor in chronic hepatitis B virus infection [2]. At present, hepatitis B accounts for the largest HCC burden globally [3]. Recently, with in-depth tumor research and rapid developments in science and technology, the early detection rate and treatment efficacy of HCC have improved [4]. With extensive studies, an increasing number of molecular mechanisms that promote HCC are being discovered, including the inactivation of tumor suppressor genes (such as p53), abnormal activation of oncogenes (such as K-ras), multiple signaling pathways (PI3K, MAPK, JAK/STAT, etc.), and abnormal regulation of epigenetic factors (such as microRNAs) are all involved in HCC development and progression [5]. However, HCC patients still have an extremely shorter survival. Thus, it is very important to explore the novel diagnosis tools, therapy strategy, and prognosis biomarkers for HCC.

Adhesion G protein-coupled receptors (GPCRs) are seven-span transmembrane receptors widely expressed in various cells that play vital roles in cell adhesion and signal transduction [6]. The epidermal growth factor seven-span transmembrane family comprises adherent GPCRs with multiple functions, constituting the largest family of cell surface proteins [7]. CD97/ADGRE5 belongs to class B GPCRs, consisting of an extracellular α-subunit and a transmembrane β-subunit [8]. CD97 is the most widely expressed member of the epidermal growth factor seven-span transmembrane family and is found on the surface of lymphocytes, monocytes, macrophages, dendritic cells, granulocytes, and smooth muscle [9]. It plays a role in cell adhesion, migration, and regulation of intercellular junctions [9]. Previous studies have investigated the relationship between CD97 expression and tumors. Ward et al. found that CD97 and lysophosphatidic acid receptor 1 heterodimerize and function synergistically, mediate Gα12/13 to increase RHO-GTP levels in thyroid and prostate cancers, and promote tumor invasion [10, 11]. Aust et al. found high expression of CD97 in gastric, pancreatic, and esophageal carcinomas [12]. Staining of CD97 was stronger in aggressive tumor cells in > 50% of gastric cancer (GC) patients, and a similar expression pattern was also observed in colorectal cancer. The high expression of CD97 was associated with a worse clinical stage and lymphatic invasion [13]. In a study on rectal adenocarcinoma, the expression of CD97 was significantly higher in patients with recurrence or metastasis than in patients without recurrence or metastasis [14]. Chidambaram et al. found that CD97 increased the migration ability of tumor cells in glioblastoma, but with no effect on proliferation, and the survival of patients with CD97 overexpression was significantly shorter than that in patients with lower expression [15]. Liu et al. found that CD97 was related to the invasion depth and TNM stage of human GC and that CD97 promoted GC cell proliferation and invasion via the MAPK signaling pathway, mediated by exosomes [16, 17]. Yin et al. found that the synergy between CD97 and GPCR kinase 6 (GRK6) promoted epithelial–mesenchymal transition by regulating the expression of downstream MMP2/MMP9, leading to metastasis of HCC [18]. The function of CD97 in tumors remains unknown. Although many studies have shown that CD97 plays a key role in tumor invasion, metastasis, and angiogenesis, the specific regulatory mechanisms remain unclear. With this background, it is especially appealing to explore the role of CD97 in the pathogenesis and development of tumors.

Here, we found that CD97 was correlated with immune cell infiltration and can be used as a prognostic marker in HCC. First, we revealed that CD97 and genes co-expressed with CD97 were abnormally expressed and related to prognosis in HCC. Second, we analyzed the signaling pathways that CD97 is involved in. Furthermore, we showed the relationships among CD97, tumor-infiltrating immune cells (TIICs), and immune-related genes. This study aims to provide a new perspective on the underlying mechanisms of hepatocellular carcinogenesis and to help identify new potential targets for diagnosing and treating HCC.

## Materials and methods

### CD97 gene expression analysis

We used the pan-cancer analysis in the TNMplot database to evaluate the mRNA levels of CD97 in various tumors. The TNMplot database [19] is a collection of 56,938 high-quality tissue samples, including RNA-seq data from the Genotype-Tissue Expression (GTEx), The Cancer Genome Atlas (TCGA), and the Therapeutically Applicable Research to Generate Effective Treatments (TARGET) databases, as well as GeneChip data from the Gene Expression Omnibus (GEO) database. Next, we selected two datasets (GSE14323 [20] and GSE64041 [21]) containing HCC and normal hepatic tissue gene expression data from the GEO database [22]. Differences in CD97 mRNA expression between HCC and normal hepatic tissues were analyzed, and plots were created using GraphPad Prism (version 8.0.0) software. We also used the Human Protein Atlas (HPA) database to analyze differences in CD97 protein expression between HCC and normal hepatic tissues.

### Immunohistochemistry (IHC) staining and scoring

For IHC analysis of CD97 protein expression, the study enrolled 20 patients with HCC from January 2021 to December 2021 at The Affiliated Calmette Hospital of Kunming Medical University (Yunnan, China). For IHC staining, thick serial sections of paraffin-embedded tumor fragment from surgically resected HCC were used to prepare the slides. Antigen retrieval was performed with citrate antigen retrieval solution, and blocked with the endogenous peroxidase enzyme blocking buffer and normal goat serum, CD97 rabbit monoclonal antibody (#GTX108192; GeneTex) at a dilution of 1:200 was incubated with the slides. The slides were observed and photographed using an inverted microscope. The IHC staining score of CD97 in each sample was as follows: one slide was evaluated for each sample, and five regions were randomly selected for each slide for scoring and the average is obtained; the staining extent score was on a scale of 0–4, corresponding to the percentage of stained tumor cells (0%, 1–5%, 6–25%, 26–75%, and 76–100%, respectively), while staining intensity was scored as negative (score = 0), weak (score = 1), moderate (score = 2), and strong (score = 3); the final IHC staining score of CD97 was calculated by multiplying the staining extent score with the intensity score. IHC staining was assessed by two independent pathologists, and a plot was created using GraphPad Prism (version 8.0.0) software.

### Correlation analysis between CD97 and clinicopathological features

We downloaded the clinicopathological data of primary HCC patients from the UCSC Xena browser [23]. The HCC cohort in TCGA contains 115 clinical parameters. We selected age, sex, tumor grade, invasion depth, lymph node metastasis, distant metastasis, TNM stage, and survival status as the clinicopathological parameters. After excluding the HCC patients with missing clinicopathological data, we classified the remaining 365 patients into two groups according to the median expression level of CD97: high and low CD97 expression groups. The chi-square test was used to analyze the correlations between CD97 expression and clinicopathological parameters (SPSS version 25.0 software).

### Prognostic analysis of CD97 expression in HCC

The Kaplan–Meier plotter (KM plotter) online database [24] was used to evaluate the prognostic value of specific genes in HCC patients (*n* = 364). UALCAN is a web analysis tool [25] of TCGA that is also used to analyze the prognostic impact of individual genes. We used the KM plotter and UALCAN databases to analyze the relationship between CD97 expression and prognosis in HCC. Furthermore, we used a KM plotter to analyze the clinicopathological features of patients with HCC, and these were mapped using the “forestplot” package (version 2.0.1).

### Significant prognostic marker analysis

We downloaded prognostic data of patients with primary HCC from the UCSC Xena browser. We used SPSS (version 25.0) software to determine the independent prognostic factors by univariate and multivariate Cox regression analyses. The results are presented as hazard ratios (HRs), 95% confidence intervals (CIs), and *P*-values.

### Gene set enrichment analysis

We downloaded processed data of patients with HCC from the UCSC Xena browser and divided the patients into high and low CD97 expression groups according to the median expression of CD97. Gene set enrichment analysis (GSEA) (version 4.1.0) software was used to identify the enriched Kyoto Encyclopedia of Genes and Genomes (KEGG) pathways in both groups. In the GSEA analysis, the number of genes parameters was 1000, and the nominal *P*-value, false discovery rate, and normalized enrichment score were used to evaluate the enriched pathways.

### Profiling of genes positively related with CD97

To investigate the molecular mechanism of CD97, we used the UALCAN web analysis tool to screen genes positively related with CD97 and select CD97-associated proteins. We selected the top six genes most positively correlated with CD97 for further in-depth research. The relationship between these genes was verified using the Tumor Immune Estimation Resource (TIMER) database [26]. Moreover, we used TNMplot and UALCAN web analysis tools to explore expression differences among genes positively related with CD97. Finally, we used the KM plotter and UALCAN to analyze the prognostic value of the genes positively related with CD97.

### Correlation analysis between CD97 or CD97 co-expressed gene expression and immune infiltrating cells

We used the TIMER database to analyze TIICs, including CD8+ T cells, CD4+ T cells, B cells, macrophages, neutrophils, and dendritic cells. We also explored the relationships of the expression of CD97 and its co-expressed genes (IQGAP1, GMIP, MOBKL2A, ARPC2, CD68, and FAM102B) with TIICs. Furthermore, we used the CIBERSORT website [27] to identify the LM22 gene in the HCC samples (including 22 immune cell types) to explore the proportion of immune cells in the HCC high and low CD97 expression groups further. Next, we used the “vioplot” package (version 0.3.7) to create the figures. The TIMER database was used to analyze the relationships between CD97 and gene markers of M1 macrophages, M2 macrophages, and tumor-associated macrophages (TAMs) in HCC.

### Correlation analysis between CD97 and tumor immunity-related parameters

We evaluated changes in the tumor microenvironment (TME) in HCC based on the CIBERSORT analysis results and used the “ggplot2” package (version 3.3.3), “heatmap” package (version 1.0.12), and “corrplot” package (version 0.92) in the RStudio (version 3.5.2) to generate the corresponding images. Next, we used the TIMER database to analyze the relationship between the expression of CD97 and that of five immune checkpoints (TOX, PD-L1, PD-L2, CTLA4, and PD-1). We also analyzed the relationship between CD97 copy number changes and the level of immune cell infiltration in HCC. Finally, we used the cBioportal database [28] to analyze the relationship between CD97 copy number alternations and mRNA level changes.

### Data analysis

We used RStudio (version 3.5.2) and SPSS (version 25.0) for the statistical analyses, and the values are presented as means ± standard deviation. We used independent and paired sample *t*-tests to analyze differences in CD97 mRNA expression between HCC and normal hepatic tissues based on the TNMplot and GEO databases. The Pearson chi-squared test was used to analyze the association between CD97 expression and characteristic clinical variables. We then used the KM plotter and UALCAN database to generate survival curves. Univariate and multivariate analyses were performed using Cox proportional hazards regression. Statistically significant differences were defined by *P* < 0.05.

## Results

### Elevated expression of CD97 in HCC

**Fig. 1:**
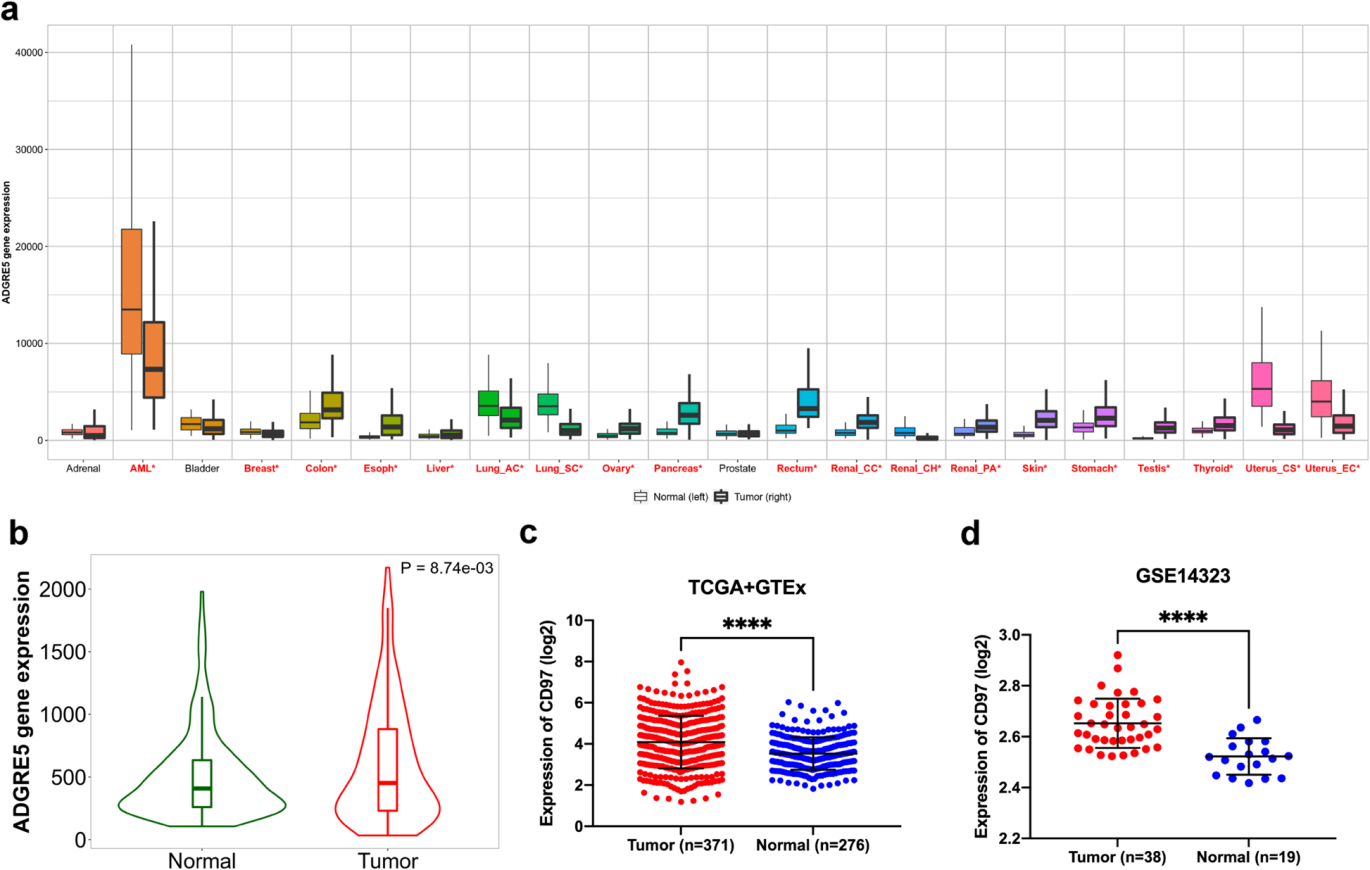
CD97 mRNA expression in HCC. **a** CD97 mRNA levels in different tumors analyzed in TNMplot. **b** CD97 mRNA levels in HCC tissues and normal hepatic tissues in TNMplot. CD97 mRNA levels in HCC tissues and normal hepatic tissues in the GSE14323 (**c**) and GSE64041 (**d**) datasets. *P < 0.05, **P < 0.01, and ***P < 0.001

**Fig. 2:**
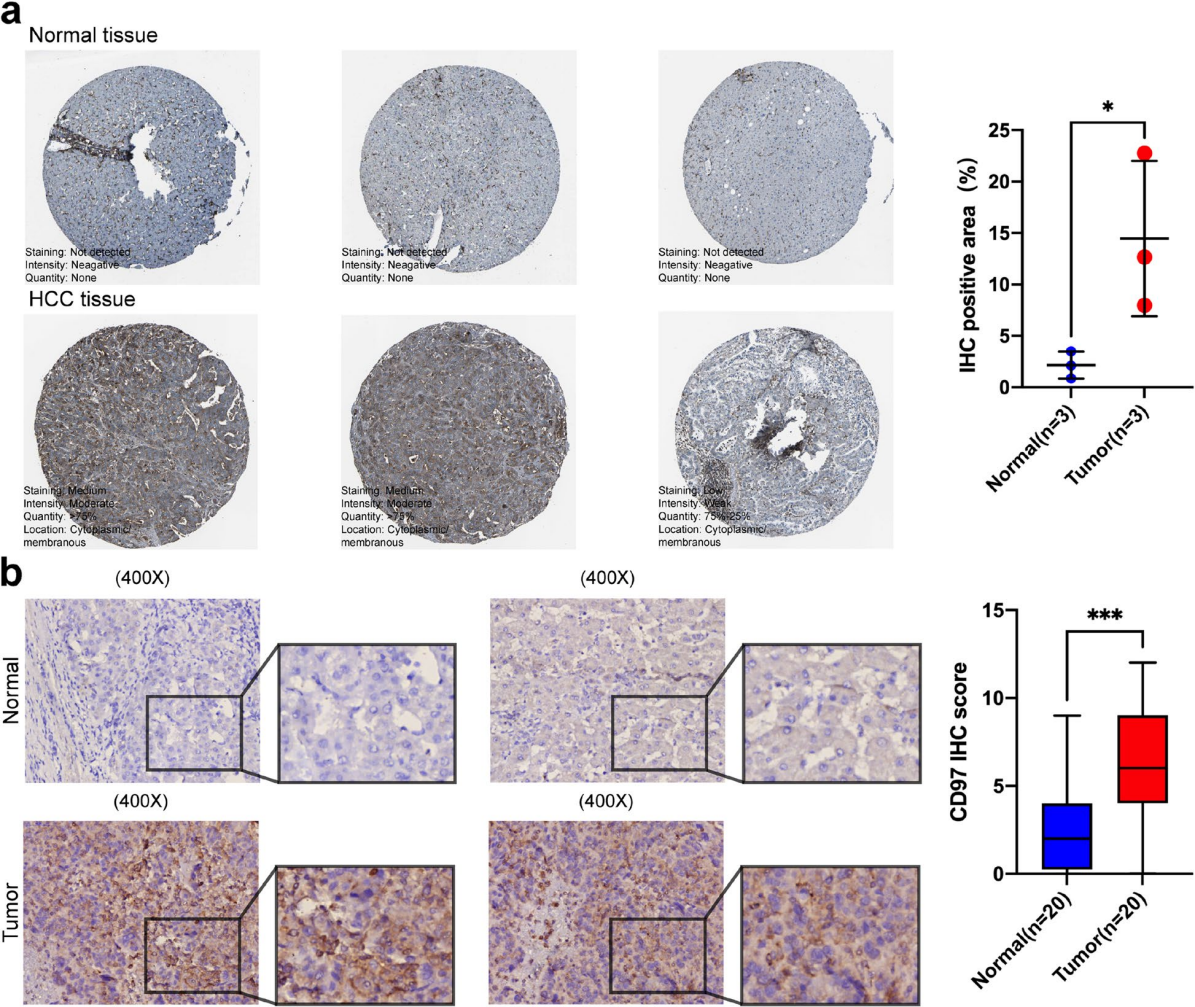
CD97 protein levels in HCC. Based on the HPA database (**a**) and our study (**b**), IHC staining analysis of CD97 protein levels in patients with HCC. Corresponding images and statistics of CD97 levels in adjacent normal hepatic tissues and HCC tissues are shown. *P < 0.05 and ***P < 0.001

We evaluated CD97 mRNA levels in different tumors by analyzing RNA-seq and GeneChip data in the TNMplot database (Fig. 1a), and these results indicate that CD97 is abnormally expressed in various tumor tissues. We showed that CD97 mRNA expression was significantly higher in HCC tissue than in normal hepatic tissue (Fig. 1b). Next, we evaluated CD97 mRNA expression in HCC and normal hepatic tissues using the GEO database and found higher expression in HCC tissue than in normal hepatic tissue (Fig. 1c, d). Moreover, IHC data from the HPA database showed a significantly higher CD97 protein level in HCC than normal hepatic tissues (*P* = 0.0497; Fig. 2a). Similarly, in our study, the IHC staining score of CD97 in HCC tissues was significantly higher than adjacent normal hepatic tissues (*P* < 0.001; Fig. 2b). These results suggest higher CD97 expression in HCC than normal hepatic tissues.

### Relationship between CD97 expression and clinicopathological features in HCC patients

We assessed the relationship between CD97 mRNA expression and clinicopathological features of HCC patients by using HCC data from TCGA. Based on the median CD97 mRNA level, we assigned 183 HCC patients to the CD97 high expression group and 182 patients to the CD97 low expression group. As shown in Table 1, there were no significant differences in clinicopathological characteristics, including survival status (*P* = 0.052), invasion depth (*P* = 0.099), and TNM stage (*P* = 0.099), between the two groups.

**Table 1:** Relationship between CD97 expression level and clinicopathological variables in HCC patients

Classification	Total	CD97 expression		***Χ***^**2**^	***P-***value
		High	Low		
**Age**				1.732	0.188
<60	169	91	78		
≥60	196	92	104		
**Sex**				1.902	0.168
Male	247	130	117		
Female	118	53	65		
**Histologic grade**				1.172	0.760
G1	55	25	30		
G2	177	87	90		
G3	121	65	56		
G4	12	6	6		
**Invasion depth**				6.269	0.099
T1	181	79	102		
T2	91	52	39		
T3	80	44	36		
T4	13	8	5		
**Lymph node metastasis**				0.000	1
N0–Nx	361	181	180		
N1	4	2	2		
**Distant metastasis**				0.247	0.619
M0–Mx	361	180	181		
M1	4	3	1		
**TNM stage**				6.283	0.099
I	179	78	101		
II	89	51	38		
III	89	49	40		
IV	8	5	3		
**Status**				3.778	0.052
Alive	239	111	128		
Dead	126	72	54		

*P*-value < 0.05, statistically significant

### The prognostic value of CD97 expression in HCC

First, we analyzed the association between CD97 expression and the prognosis of HCC patients. The prognosis of HCC patients with high CD97 expression was significantly worse in the UALCAN (*P* < 0.001; Fig. S1a) and KM plotter (HR = 1.8, *P* < 0.001; Fig. S1b) databases compared with the patients with low expression. Kaplan–Meier survival analysis showed that male sex (HR = 2.37, *P* < 0.001), Asian ethnicity (HR = 3, *P* < 0.001), alcohol consumption (HR = 1.96, *P* = 0.036), no alcohol consumption (HR = 1.89, *P* = 0.0068), and no hepatitis viral infection (HR = 1.86, *P* = 0.0068) were associated with a poor prognosis in the high CD97 expression group (Fig. S1c). The prognosis of HCC patients with high CD97 expression was significantly worse at TNM stages III and IV, pathological grades II and III, and American Joint Committee on Cancer stage III (Fig. S1c). These results indicate that the prognosis of HCC patients with high CD97 expression is correlated with clinicopathological features, especially for those with advanced HCC. Furthermore, Cox univariate survival analysis showed that grade (*P* < 0.001), invasion depth (*P* < 0.001), distant metastasis (*P* = 0.026), TNM stage (*P* < 0.001), and CD97 expression (*P* = 0.048) were significantly associated with the prognosis of HCC patients (Table 2). Cox multivariate survival analysis revealed that TNM stage and CD97 expression were independent risk factors for adverse prognosis in HCC (all *P* < 0.05; Table 3). In summary, our results suggest that CD97 expression predicts an adverse outcome and acts as an independent prognostic marker in HCC.

**Table 2:** Univariate analysis of the prognostic factors in HCC patients using a Cox regression model

Parameters	Univariate analysis		
	Hazard ratio	95% CI	***P***-value
Ages, year (≥60 vs. < 60)	1.195	0.839–1.702	0.323
Sex (male vs. female)	1.198	0.834–1.720	0.328
Grade (G3–G4 vs. G1–G2)	2.594	1.819–3.701	**<0.001**
Invasion depth (T3/T4 vs. T1/T2)	2.539	1.767–3.647	**<0.001**
Lymph node metastasis (N1 vs. N0–Nx)	1.869	0.458–7.543	0.368
Distant metastasis (M1 vs. M0–Mx)	3.681	1.166–11.615	**0.026**
TNM stage (III–IV vs. I–II)	2.566	1.801–3.657	**<0.001**
CD97 expression (high vs. low)	1.428	1.003–2.034	**0.048**

*CI* confidence interval. *P*-value < 0.05, statistically significant

**Table 3:** Multivariate analysis of the prognostic factors in HCC patients using a Cox regression model

Parameters	Multivariate analysis		
	Hazard ratio	95% CI	***P-***value
Ages, year (≥60 vs. < 60)	1.189	0.828–1.708	0.349
Sex (male vs. female)	1.124	0.773–1.636	0.540
Grade (G3–G4 vs. G1–G2)	1.183	0.820–1.708	0.369
Lymph node metastasis (N1 vs. N0–Nx)	1.019	0.242–4.296	0.980
Distant metastasis (M1 vs. M0–Mx)	1.709	0.517–5.655	0.380
TNM stage (III–IV vs. I–II)	2.498	1.735–3.597	**<0.001**
CD97 expression (high vs. low)	1.445	1.010–2.068	**0.044**

*CI* confidence interval. *P*-value < 0.05, statistically significant

### CD97-related signaling pathways in HCC identified by GSEA

**Fig. 3:**
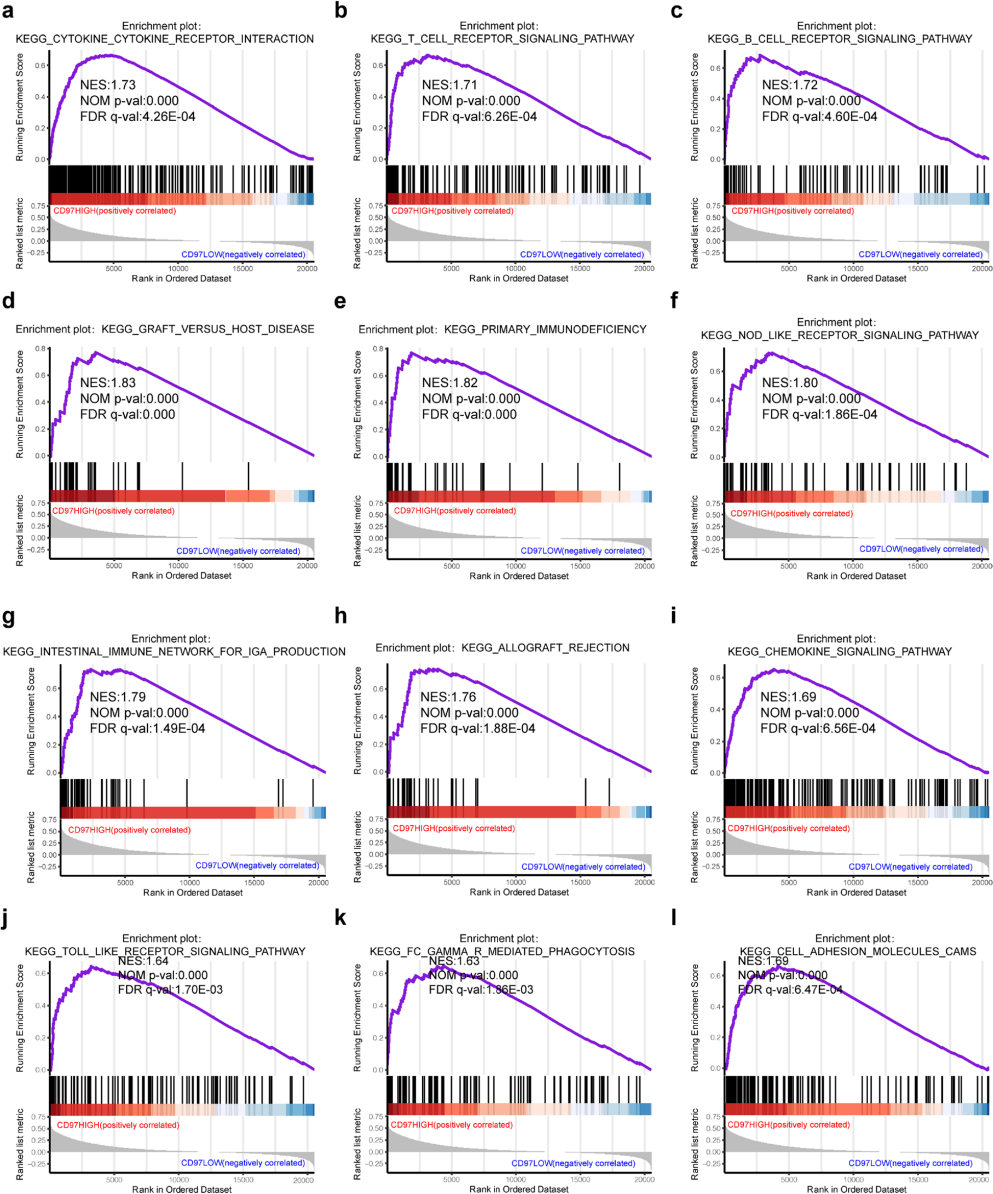
GSEA pathways enriched in HCC samples with high CD97 expression. The GSEA results showed that the terms “cytokine receptor interaction” (**a**), “T cell receptor signaling pathway” (**b**), “B cell receptor signaling pathway” (**c**), “graft versus host disease” (**d**), “primary immunodeficiency” (**e**), “NOD-like receptor signaling pathway” (**f**), “intestinal immune network for IgA production” (**g**), “allograft” (**h**), “chemokine signaling pathway” (**i**), “toll-like receptor signaling pathway” (**j**), “FC gamma R mediated phagocytosis” (**k**), and “cell adhesion molecules CAMs” (**l**) were enriched in HCC samples with high CD97 expression. NES, normalized enrichment score

We explored the molecular mechanism of CD97 in the development of HCC using GSEA and found 131 KEGG signaling pathways associated with the high CD97 expression group, of which 19 were significantly enriched in the high CD97 expression group (nominal *P* < 0.05, false discovery rate < 0.1, and normalized enrichment score > 1.6; Table S1). These signaling pathways were positively correlated with immunity and inflammation, including “cytokine receptor interaction,” “T cell receptor signaling pathway,” “B cell receptor signaling pathway,” “graft versus host disease,” “primary immunodeficiency,” “NOD-like receptor signaling pathway,” “intestinal immune network for IgA production,” “allograft,” “chemokine signaling pathway,” “toll-like receptor signaling pathway,” and “FC gamma R mediated phagocytosis,” and to cell adhesion, including “cell adhesion molecules CAMs” (Fig. 3).

### Co-expression analysis of CD97 in HCC

**Fig. 4:**
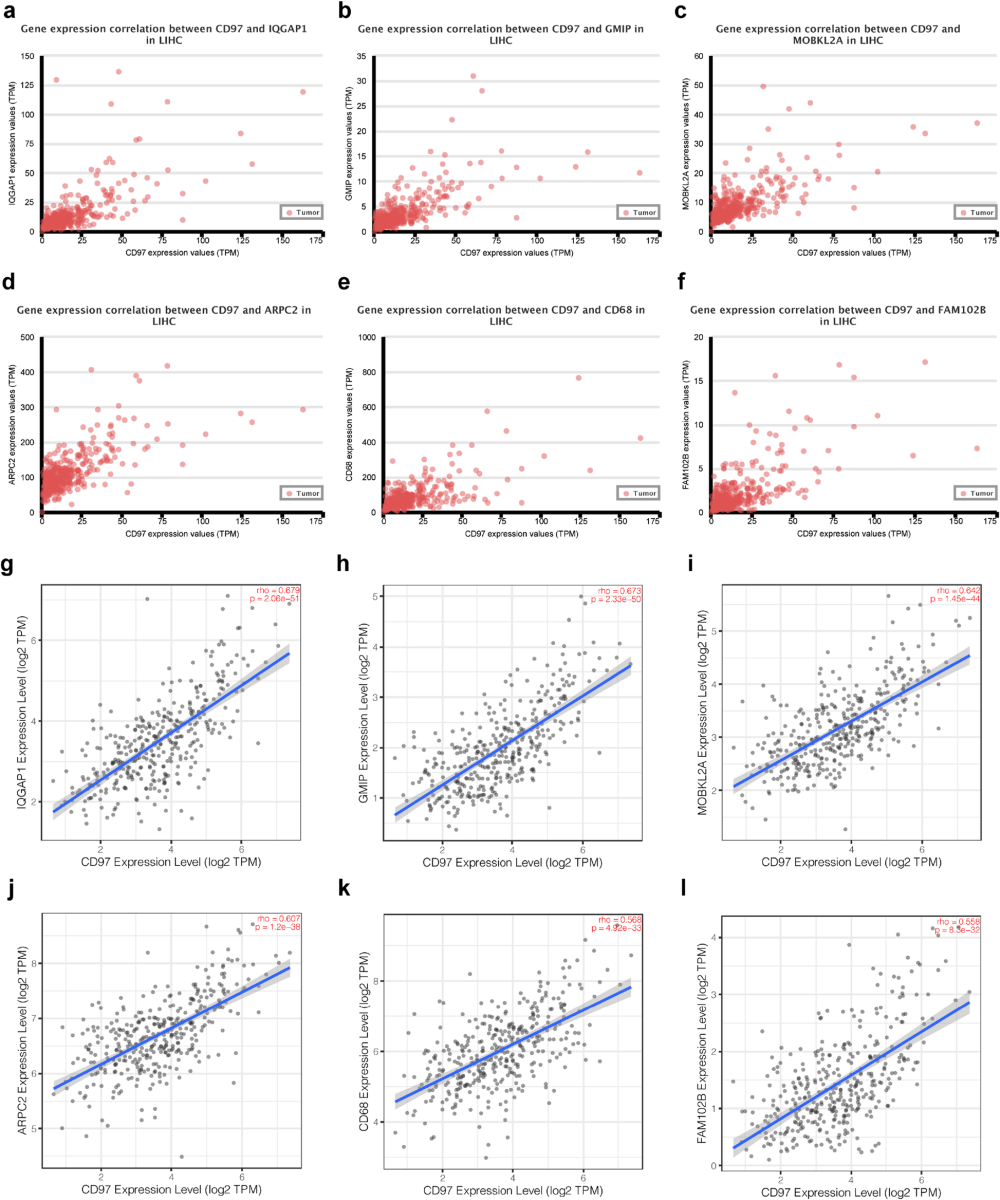
The top six genes co-expressed with CD97 in HCC. **a**–**f** The genes co-expressed with CD97 in HCC (*r* ≥ 0.66) were assessed using the UALCAN database. **g**–**l** CD97 was positively correlated with IQGAP1 (*r* = 0.679, *P* < 0.001), GMIP (*r* = 0.673, *P* < 0.001), MOBKL2A (*r* = 0.642, *P* < 0.001), ARPC2 (*r* = 0.607, *P* < 0.001), CD68 (*r* = 0.568, *P* < 0.001), and FAM102B (*r* = 0.558, *P* < 0.001) in HCC in the TIMER database

**Fig. 5:**
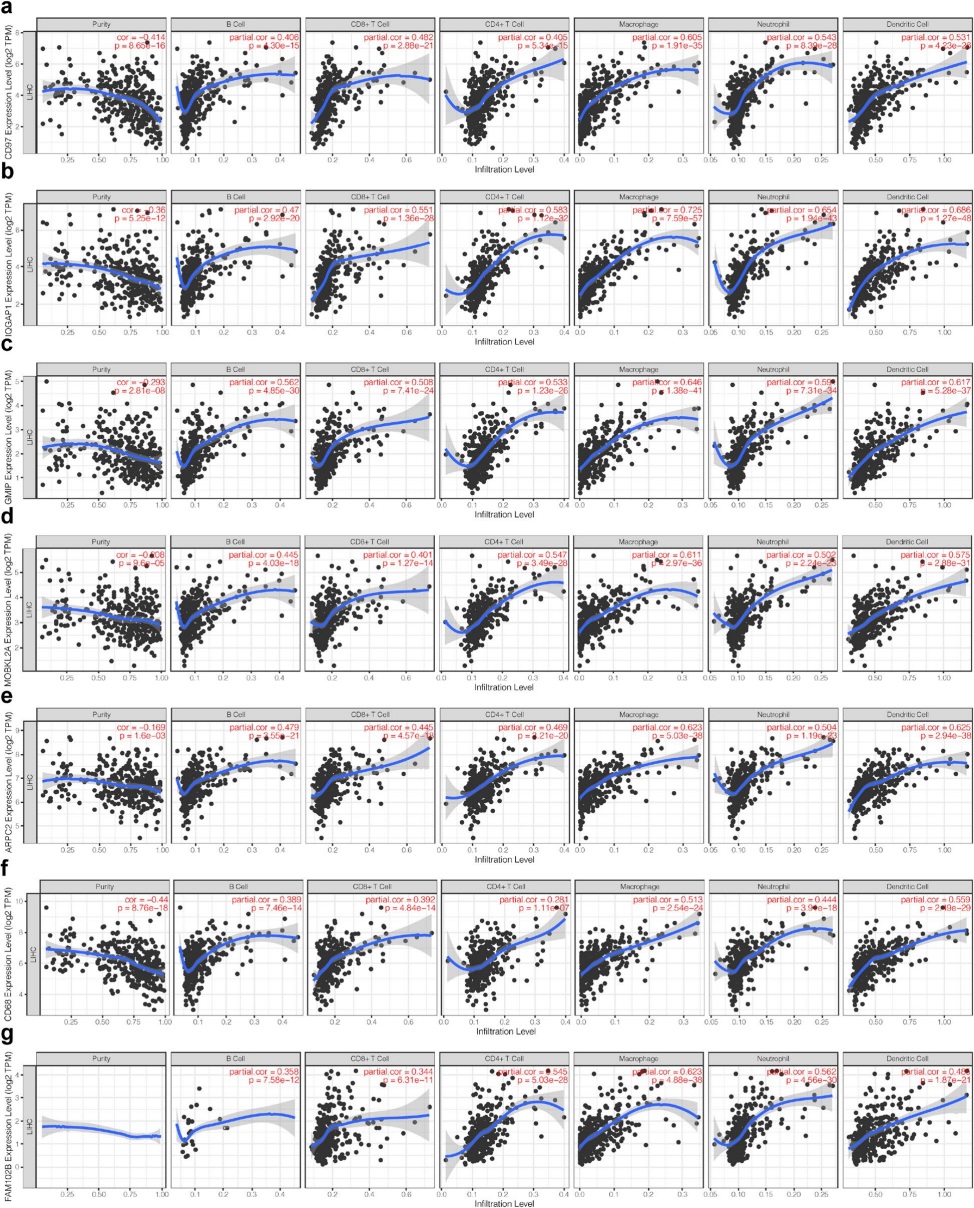
Correlations of CD97 and its co-expressed genes with immune cell filtration levels in HCC. Correlations of CD97 (**a**), IQGAP1 (**b**), GMIP (**c**), CDH11 (**d**), MOBKL2A (**e**), ARPC2 (**f**), and FAM102B (**g**) expression with TIICs in HCC

We used the UALCAN analysis tool to mine for genes co-expressed with CD97 to further evaluate the effect of CD97 on HCC development. We selected the top six genes co-expressed with CD97 (*r* ≥ 0.66) (Fig. 4). Next, we used the TIMER database to verify the co-expression relationships between CD97 and these genes. The results showed that CD97 expression was positively related with IQGAP1 (*r* = 0.679, *P* < 0.001), GMIP (*r* = 0.673, *P* < 0.001), MOBKL2A (*r* = 0.642, *P* < 0.001), ARPC2 (*r* = 0.607, *P* < 0.001), CD68 (*r* = 0.568, *P* < 0.001), and FAM102B (*r* = 0.558, *P* < 0.001) (Fig. 5g–l). UALCAN analysis showed that only CD68 expression was decreased in HCC, while the other five genes had high expression (Fig. S2). The TNMplot database (Fig. S3) mirrored these findings. We also explored the correlations of the six genes with the prognosis of HCC patients using the UALCAN database and found that HCC patients with high GMIP, MOBKL2A, ARPC2, CD68, or FAM102B expression had a significantly worse prognosis (Fig. S4). The KM plotter database showed the same results: patients with high GMIP (HR = 1.46, *P* = 0.031), MOBKL2A (HR = 1.49, *P* = 0.022), ARPC2 (HR = 2.03, *P* = 0.002), CD68 (HR = 1.44, *P* = 0.046), or FAM102B (HR = 1.57, *P* = 0.011) expression had a shorter survival (Fig. S5). These results suggested that CD97 and genes positively related with CD97 are associated with the development and poor prognosis of HCC.

### Significant correlations of CD97 and its co-expressed genes with TIICs in HCC

We used the TIMER database to explore the relationship between CD97 expression and TIICs in the HCC microenvironment. The expression of CD97 was positively correlated with different TIICs, including B cells (*r* = 0.406, *P* < 0.001), CD8+ T cells (*r* = 0.482, *P* < 0.001), CD4+ T cells (*r* = 0.405, *P* < 0.001), neutrophils (*r* = 0.543, *P* < 0.001), and dendritic cells (*r* = 0.531, *P* < 0.001), and was significantly related to macrophages (*r* = 0.605, *P* < 0.001) (Fig. 5a). Further analysis of the relationships between CD97-co-expressed genes and TIICs in HCC showed that IQGAP1, GMIP, MOBKL2A, ARPC2, CD68, and FAM102B were significantly positively correlated with TIICs, especially macrophages (all *r* ≥ 0.281, *P* < 0.001; Fig. 5b–g). These results suggest that CD97 and its co-expressed genes are involved in the immune response in the TME by affecting TIICs, especially macrophages.

**Fig. 6:**
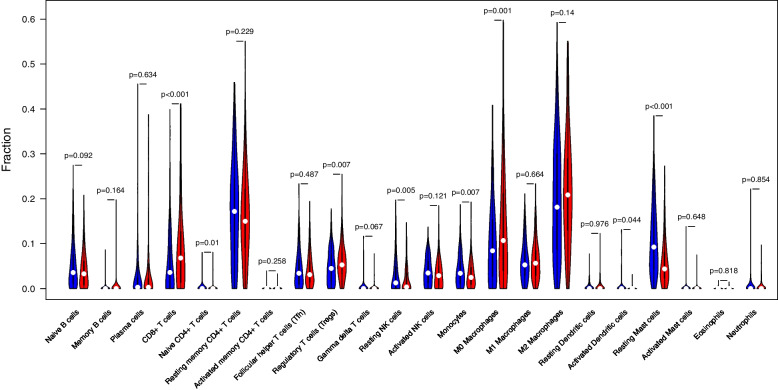
Violin plots visualizing the distributions of TIICs according to CD97 expression using the CIBERSORT database. Blue and red represent the CD97 low and high expression groups, respectively. There were significant differences in the numbers of CD8+ T cells (*P* < 0.001), naive CD4 + T cells (*P* = 0.01), regulatory T cells (*P* = 0.007), resting NK cells (*P* = 0.005), monocytes (*P* = 0.007), M0 macrophages (*P* = 0.001), activated dendritic cells (*P* = 0.044), and resting mast cells (*P* < 0.001)

CIBERSORT analysis showed that CD97 expression was significantly associated with TIICs, including CD8+ T cells (*P* < 0.001), naive CD4 + T cells (*P* = 0.01), regulatory T cells (*P* = 0.007), resting NK cells (*P* = 0.005), monocytes (*P* = 0.007), M0 macrophages (*P* = 0.001), activated dendritic cells (*P* = 0.044), and resting mast cells (*P* < 0.001) (Fig. 6). In short, CD97 and its co-expressed genes showed close correlations with TIICs in HCC.

### CD97 is associated with M2 macrophage polarization

**Fig. 7:**
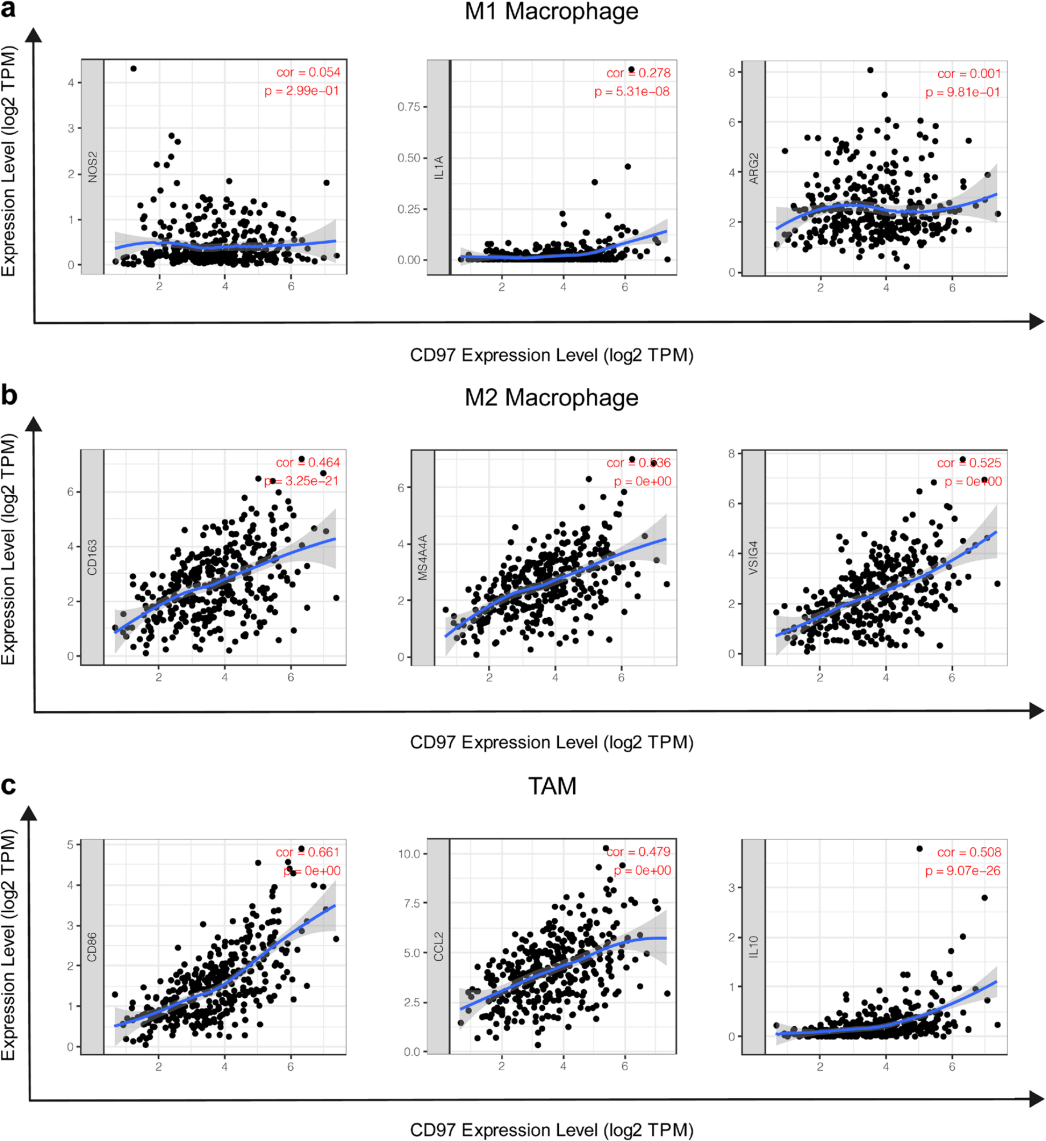
Correlation of CD97 expression with macrophage polarization in HCC. **a** Correlations between CD97 expression and gene markers of M1 macrophages (NOS2, IL1A, and ARG2). **b** Correlations between CD97 expression and gene markers of M2 macrophages (CD163, MS4A4A, and VSIG4). **c** Correlations between CD97 expression and gene markers of TAM (CD86, CCL2, and IL10)

There was a significant positive correlation between CD97 and the infiltration level of macrophages (Fig. 5a). We explored the associations between CD97 expression and expression of markers of M1 macrophages, M2 macrophages, and TAMs in HCC using the TIMER database to determine the relationships between CD97 and the different macrophage subtypes. We found that the expression of the M1 macrophage gene marker IL1A (*r* = 0.278, *P* < 0.001) was significantly but weakly correlated with the expression of CD97 (Fig. 7a), while that of the M2 macrophage gene markers CD163 (*r* = 0.464, *P* < 0.001), MS4A4A (*r* = 0.536, *P* < 0.001), and VSIG4 (*r* = 0.525, *P* < 0.001) and the TAM markers CD86 (*r* = 0.661, *P* < 0.001), CCL2 (*r* = 0.479, *P* < 0.001), and IL10 (*r* = 0.508, *P* < 0.001) showed stronger positive correlations with CD97 expression (Fig. 7b, c). These findings suggest that high CD97 expression promotes M2 macrophage polarization and differentiation into TAMs, contributing to hepatocellular carcinogenesis.

### CD97 acts as an immune-related gene in HCC

**Fig. 8:**
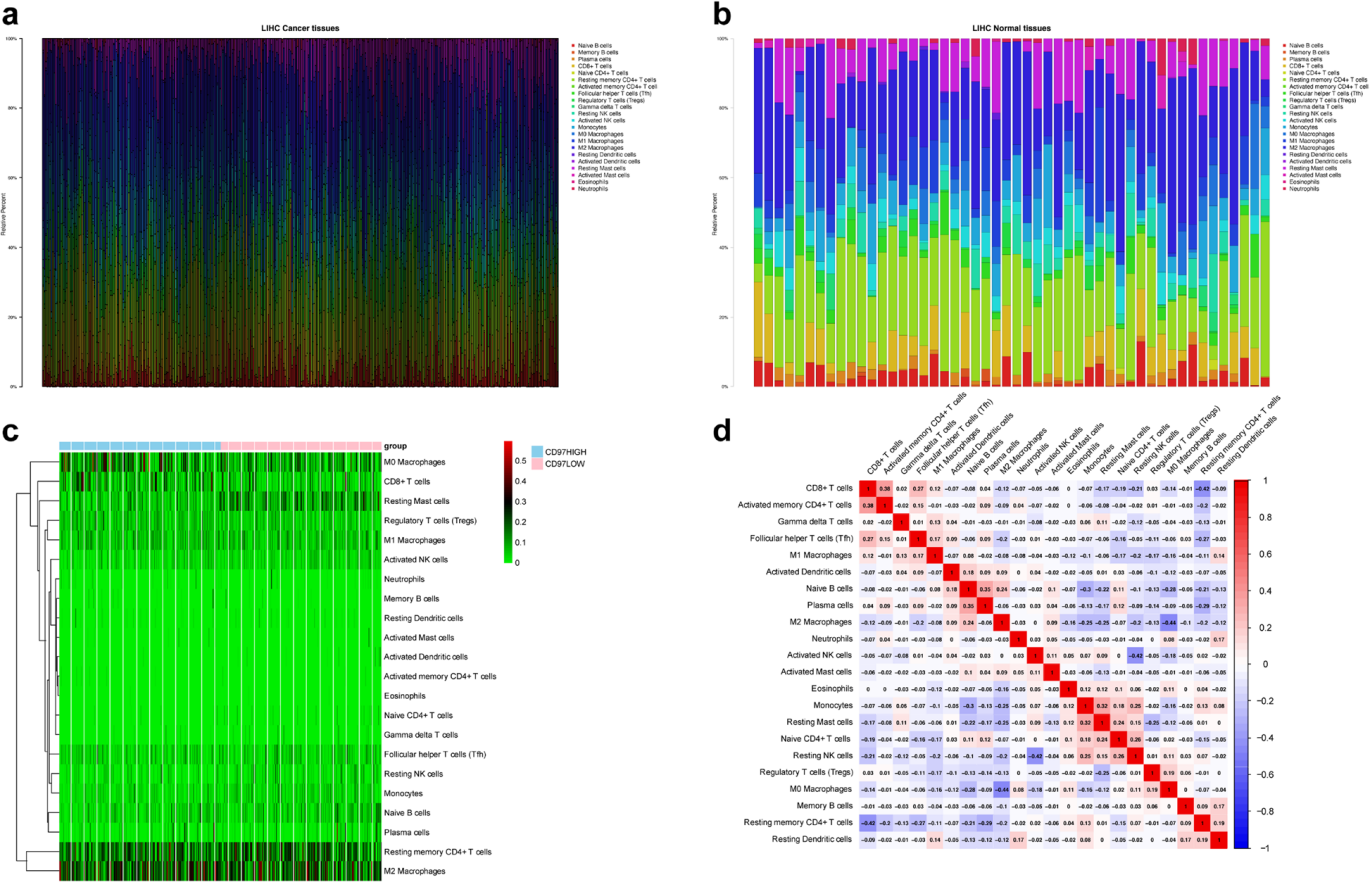
Landscape of immune cell infiltration in HCC by CIBERSORT. **a**, **b** The composition of 22 types of immune cells in HCC and normal hepatic tissues. **c** Heatmap showing the differences in the infiltration levels of 22 types of immune cells between the CD97 high and low expression groups in HCC. **d** Correlation heatmap visualizing the correlations among 22 types of immune cells in HCC

**Fig. 9:**
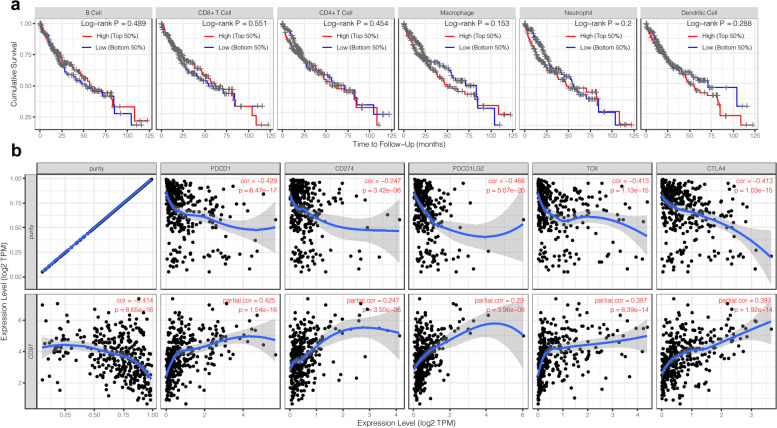
Correlation analysis between CD97 expression and tumor immunity-related parameters. The associations between immune cells and prognosis in the TIMER database (**a**). The associations between CD97 and immune checkpoint expression (**b**)

**Fig. 10:**
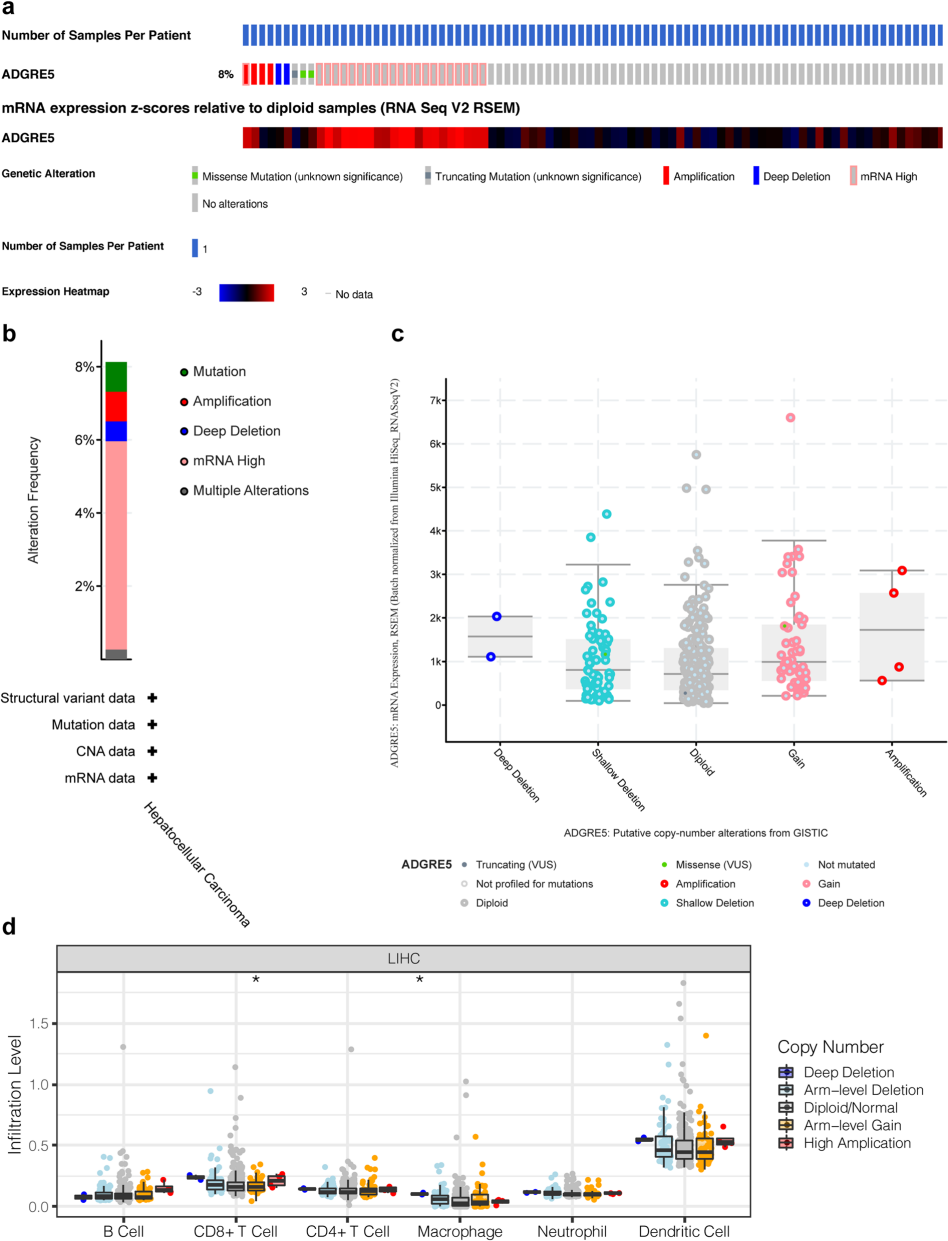
The levels of CD97 and immune cell infiltration were associated with CD97 copy number alternations. **a**–**c** The relationship between CD97 copy number and its mRNA level. **d** the relationships between CD97 copy number and immune cell infiltration levels, *P < 0.05

We used the CIBERSORT algorithm to evaluate immune cell infiltration in the TME of HCC. We obtained data from 373 primary HCC and 50 normal hepatic samples from TCGA and found significant differences in the proportions of TIICs between HCC and normal hepatic tissues (Fig. 8a, b). There was an apparent discrepancy in the proportion of TIICs between the CD97 high and low expression groups in HCC (Fig. 8c). A heatmap showed that the proportions of different TIICs in HCC were strongly correlated with each other. There were significant negative correlations between M0 and M2 macrophages (*r* = −0.44), between CD8+ T cells and resting memory CD4+ T cells (*r* = −0.42), and between resting and activated NK cells (*r* = −0.42) and a significant positive correlation between CD8+ T cells and activated memory CD4+ T cells (*r* = 0.38) (Fig. 8d). We used the TIMER database to evaluate the relationships between the proportions of diverse types of TIICs and prognosis in HCC but found no significant associations (Fig. 9a). We also analyzed the relationship between CD97 and the immune checkpoint genes TOX, PD-L1 (or CD274), PD-L2 (or PDCD1LG2), CTLA4, and PD-1 (or PDCD1), which are critical targets of immunotherapy. The results showed that CD97 expression was positively correlated with the expression of the immune checkpoint genes and negatively correlated with tumor purity (Fig. 9b). In addition, we explored whether CD97 copy number alterations had a significant impact on the infiltration level of immune cells and CD97 mRNA expression (Fig. 10). Our results indicate that the copy number of CD97 may affect immune cell infiltration and thus the prognosis of HCC. Therefore, CD97 has a potential application in HCC immunotherapy.

## Discussion

HCC is a common malignant tumor of the digestive system [1]. Although there have been substantial advancements in the diagnosis and treatment of HCC over recent decades, patient prognosis remains poor [4]. Abnormal gene expression may be involved in tumor progression and is positively correlated with patient prognosis [5]. This study showed that CD97 is abnormally highly expressed in HCC tissues. Furthermore, CD97 overexpression is significantly associated with the prognosis and overall survival (OS) of HCC patients, suggesting that CD97 is an important factor that participates in HCC development. However, our knowledge of the role of CD97 molecules in HCC is limited. We aimed to comprehensively analyze the biological functions of CD97 in HCC using public databases and to reveal its related regulatory pathways.

Transcriptomic analysis of multiple databases revealed significantly higher CD97 expression in HCC tissues than in normal hepatic tissues. Furthermore, univariate and multivariate Cox survival analyses of HCC data in TCGA showed that CD97 was an independent adverse prognostic risk factor in HCC. We speculate that CD97 is highly expressed in HCC and can be used as a molecular marker for diagnosis and prognosis, but more clinical cohorts are needed for verification.

Based on the GSEA analysis, we identified 19 upregulated signaling pathways predominantly related to immune and inflammatory responses. CD97 is widely expressed in various immune cells, which plays an essential role in T cell activation [29]. The expression of CD97 is rapidly upregulated during the activation of lymphocytes and myeloid cells, which may promote adhesion and migration of these cells to inflammatory sites [8]. Kop et al. used a mouse model of collagen-induced arthritis to demonstrate that CD97 inhibitors reduce arthritis-related joint injury and inflammation by neutralizing antibodies [30]. These discoveries demonstrate that CD97 is positively correlated with immune cell infiltration. However, we need to further explore the relationship between CD97 expression and TIICs in HCC. Moreover, the significantly upregulated “cell adhesion molecules CAMs” signaling pathway participates in cell adhesion. In the current study, cell adhesion based on E-cadherin is important for maintaining epithelial integrity, which regulates the motility that spreads to the surrounding and distant tissues, affecting HCC progression [31]. CD97 has been proven to play a vital role in intercellular connections, which involves in the adhesion and migration of tumor cells and mediates the invasion of human tumors. Together, these results indicate that CD97 participates in tumorigenesis and immune-related KEGG signaling pathways. We showed that high CD97 expression in HCC predicted adverse outcomes. Therefore, CD97 may play a role in the development of HCC by affecting these signaling pathways.

Co-expression and TIMER analyses showed that CD97 expression was positively correlated with IQGAP1, GMIP, MOBKL2A, ARPC2, CD68, and FAM102B expression. These genes are involved in inflammation, the immune response, or tumorigenesis [32–36]. Genes positively related with CD97 were highly expressed in HCC tissues, except for CD68, and were associated with a poor prognosis in HCC patients, except for IQGAP1. TIICs are positively correlated with the progression and prognosis of HCC [37]. The mRNA expression levels of CD97 and its co-expressed genes were significantly and positively correlated with B cells, CD8+ T cells, CD4+ T cells, neutrophils, dendritic cells, and macrophages. At the same time, we evaluated the proportion of TIICs in HCC based on the differential expression of CD97 using CIBERSORT analysis and found significant differences in the proportions of eight types of immune cells: CD8+ T cells, naive CD4+ T cells, regulatory T cells, resting NK cells, monocytes, M0 macrophages, activated dendritic cells, and resting mast cells. The results further suggest that CD97 is strongly associated with TIICs in HCC and that CD97 and genes positively related with CD97 may be involved in the immune response to HCC development, leading to a poor prognosis.

We identified that CD97 expression in HCC was strongly associated with marker genes of M2 macrophages and TAMs, but there were only weak associations with the marker genes of M1 macrophages. These results indicate that high CD97 expression in HCC can promote the polarization of macrophages to M2 macrophages that eventually differentiate into TAMs, contributing to HCC development. The development and progression of HCC are complex and are associated with cumulative gene mutations in tumor cells but are regulated by the TME [38]. Scientific evidence shows that the TME plays a key role in tumor stimulation and progression. TAMs are vital components of the TME in HCC [38] and are composed of M1 and M2 macrophages. M1 macrophages, known as anti-tumor macrophages, activate host defenses and kill tumor cells. M2 macrophages, known as tumor-promoting macrophages, can promote tumor progression [39]. Previous studies have shown that tumor-promoting factors affect the polarization of macrophages to M2 macrophages in the TME. TAMs are similar to M2 macrophages and have similar characteristics [40]. TAMs play important roles in tumor growth by enhancing tumor cell resistance to chemotherapy and radiotherapy and promoting tumor angiogenesis (including invasion, infiltration, and metastasis) and immunosuppression [40]. Therefore, we speculate that high CD97 expression promotes the differentiation of macrophages into TAMs, accelerating their progression and leading to a poor prognosis in HCC patients.

There was a significant positive correlation between the expression of CD97 and immune checkpoint genes such as TOX, PD-L1, PD-L2, CTLA4, and PD-1. Immune checkpoint inhibitors have made significant breakthroughs in cancer treatment as new tumor therapies [41]. In HCC, the influence of immune checkpoints on the prognosis of patients is extensive and complex; increased expression of PD-1, PD-L1, and tumor-infiltrating lymphocytes in HCC induces immunosuppression [42]. Furthermore, high expression of CTLA-4 in HCC patients inhibits activation of T cell antigen presentation and proliferation and induces apoptosis, leading to immunosuppression and HCC development and progression [43]. Therefore, we believe that CD97 may affect the progression of HCC and the prognosis of patients by altering the expression of immune checkpoint genes such as TOX, PD-L1, PD-L2, CTLA4, and PD-1.

This study has certain limitations. First, our research data were obtained from the TCGA, GTEx, GEO, and TARGET databases, and our results must be verified using larger sample sizes. Moreover, the results obtained by the algorithm based on RNA sequences may not be accurate. Additional in vivo and in vitro experiments are required to explore the potential physiological mechanism of CD97 in HCC, including its role in tumor–immune interactions.

## Conclusion

In summary, our results suggested that CD97 has prognostic value in HCC and affects tumor immunity. Patients with high CD97 expression had poorer clinicopathological features and worse prognoses compared with patients with low expression. High CD97 expression is associated with TIICs and can promote the conversion of macrophages to TAMs. Therefore, CD97 can be used as a prognostic marker in HCC.

## Supplementary Information

**Additional file 1: Figure S1.** Prognostic value of CD97 expression in HCC. a Correlation between CD97 expression and OS in HCC patients using the UALCAN database. b Correlation between CD97 expression and OS in HCC patients using the KM plotter. c OS analysis of HCC patients using the KM plotter database. OS, overall survival.

**Additional file 2: Figure S2.** The mRNA expression of CD97 co-expressed genes in HCC using the UALCAN database. a-f the mRNA expression levels of IQGAP1, GMIP, MOBKL2A, ARPC2, CD68, and FAM102B in HCC in the UALCAN database.

**Additional file 3: Figure S3.** The mRNA expression of CD97 co-expressed genes in HCC using the TNMplot database. a-f the mRNA expression levels of IQGAP1, GMIP, MOBKL2A, ARPC2, CD68, and FAM102B in HCC in the TNMplot database.

**Additional file 4: Figure S4.** The prognostic value of CD97 co-expressed genes in HCC using the UALCAN database. a-f Correlations between OS and the mRNA levels of IQGAP1, GMIP, MOBKL2A, ARPC2, CD68, and FAM102B in HCC in the UALCAN database.

**Additional file 5: Figure S5.** The prognostic value of CD97 co-expressed genes in HCC using the KM plotter database. a-f Correlations between OS and the mRNA levels of IQGAP1, GMIP, MOBKL2A, ARPC2, CD68, and FAM102B in HCC in the KM plotter database.

**Additional file 6: Table S1.** GSEA pathways upregulated due to high expression of CD97.

## Data Availability

The raw data of this study are derived from the TCGA database (https://portal.gdc.cancer.gov/), GEO database (https://www.ncbi.nlm.nih.gov/geo/), and UCSC Xena database (http://xena.ucsc.edu), which are publicly available databases.

## Abbreviations

GPCRs: G protein-coupled receptors
HCC: Hepatocellular carcinoma
GEO: Gene Expression Omnibus
TCGA: The Cancer Genome Atlas
KM: Kaplan–Meier
GSEA: Gene Set Enrichment Analysis
TIMER: Tumor Immune Estimation Resource
GC: Gastric cancer
GRK6: G protein-coupled receptor kinase 6
EMT: Epithelial–mesenchymal transition
KEGG: Kyoto Encyclopedia of Genes and Genomes
GTEx: Genotype-Tissue Expression
TARGET: Therapeutically Applicable Research to Generate Effective Treatments
HPA: The Human Protein Atlas
HR: Hazard ratio
NES: Normalized enrichment score
TME: Tumor microenvironment
OS: Overall survival
TIICs: Tumor-infiltrating immune cells
TAMs: Tumor-associated macrophages
