# Supplementary figures and images for: CD97 serves as a novel biomarker of immune cell infiltration in hepatocellular carcinoma
Source: World J Surg Oncol. 2022 Dec 4;20:382. doi: 10.1186/s12957-022-02829-2 (PMC9721038; doi:10.1186/s12957-022-02829-2)

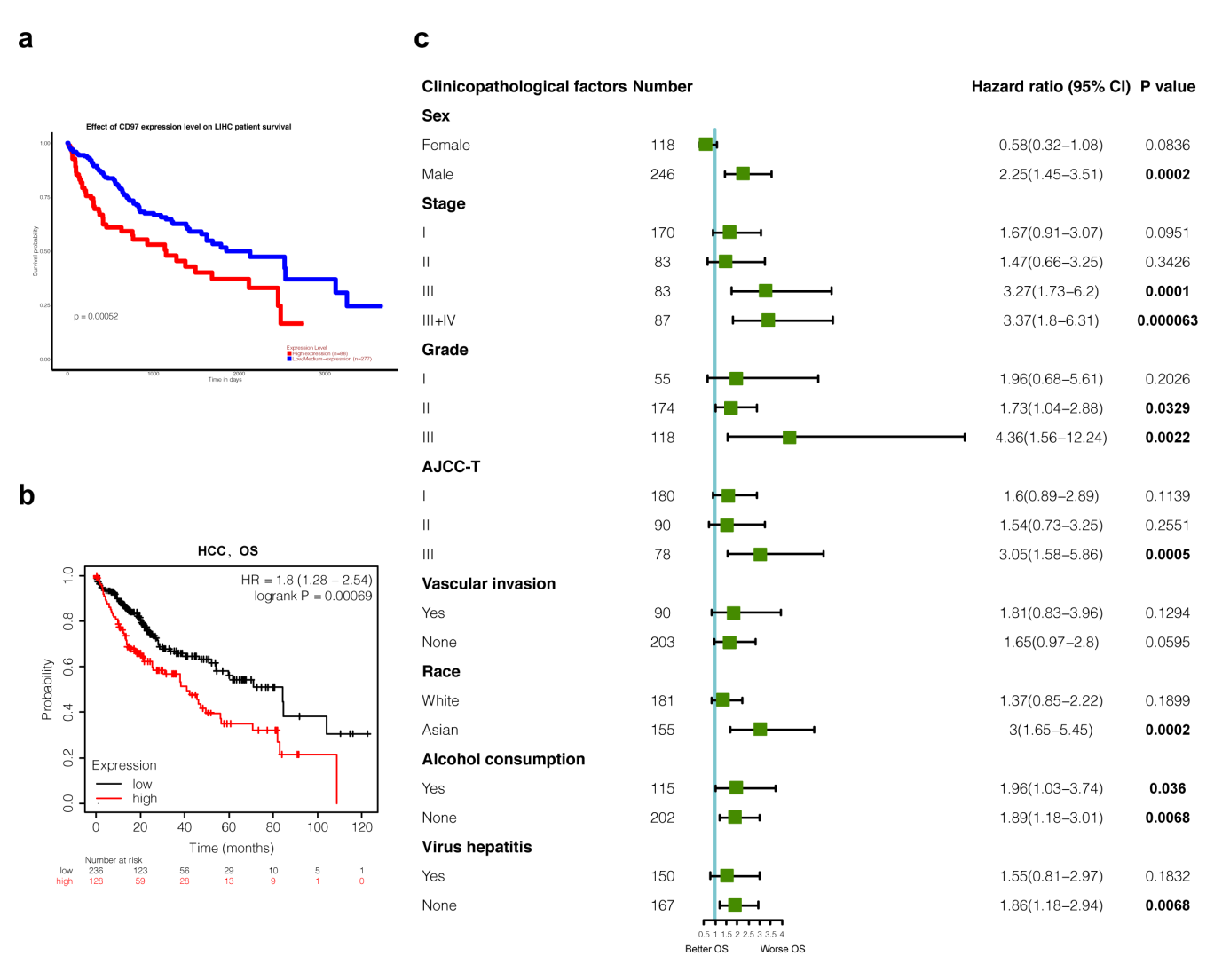

Supplement: Supplementary file 1 — Additional file 1: Figure S1. Prognostic value of CD97 expression in HCC. a Correlation between CD97 expression and OS in HCC patients using the UALCAN database. b Correlation between CD97 expression and OS in HCC patients using the KM plotter. c OS analysis of HCC patients using the KM plotter database. OS, overall survival. [file 12957_2022_2829_MOESM1_ESM.docx]

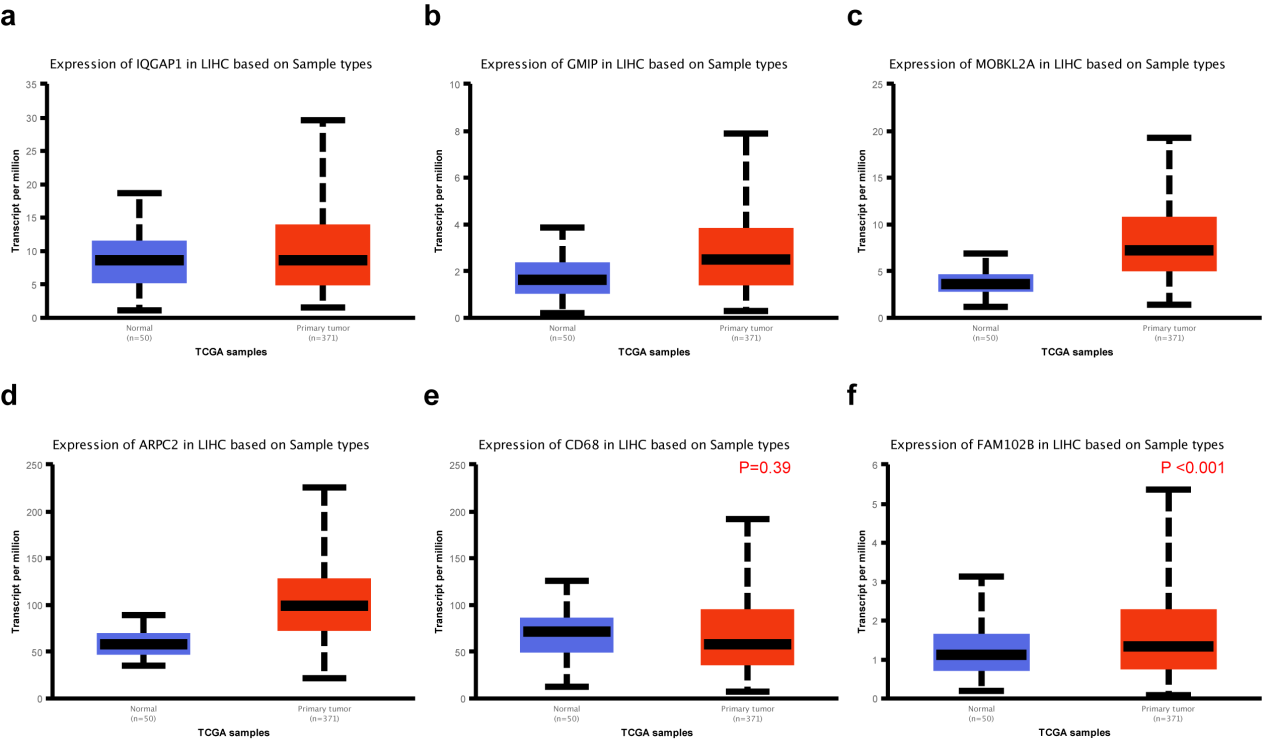

Supplement: Supplementary file 2 — Additional file 2: Figure S2. The mRNA expression of CD97 co-expressed genes in HCC using the UALCAN database. a-f the mRNA expression levels of IQGAP1, GMIP, MOBKL2A, ARPC2, CD68, and FAM102B in HCC in the UALCAN database. [file 12957_2022_2829_MOESM2_ESM.docx]

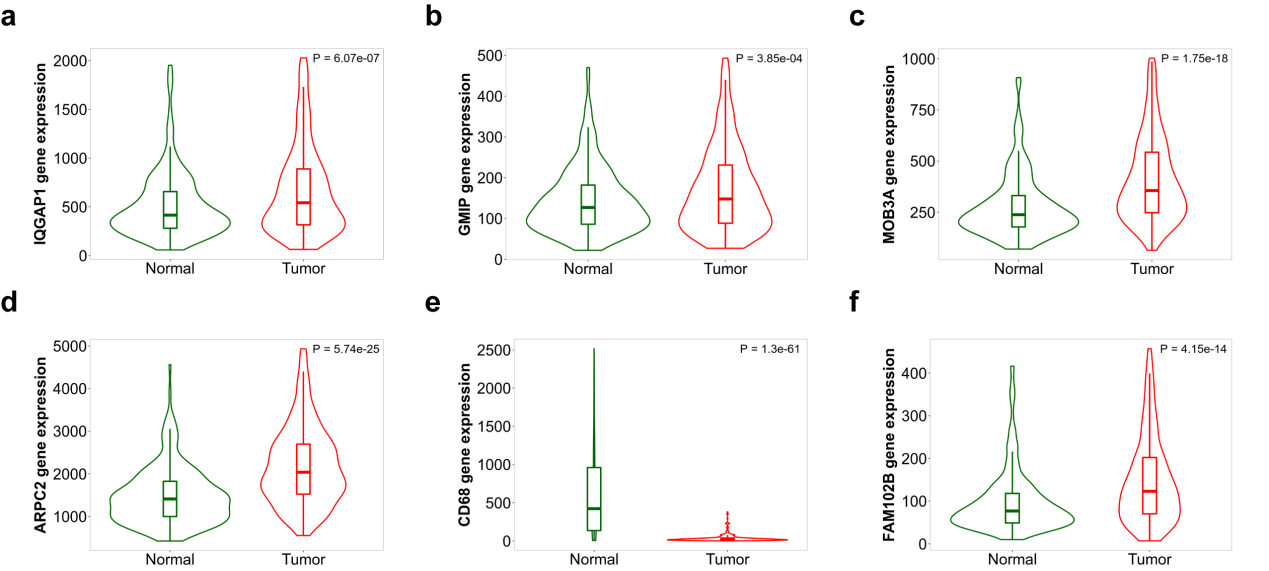

Supplement: Supplementary file 3 — Additional file 3: Figure S3. The mRNA expression of CD97 co-expressed genes in HCC using the TNMplot database. a-f the mRNA expression levels of IQGAP1, GMIP, MOBKL2A, ARPC2, CD68, and FAM102B in HCC in the TNMplot database. [file 12957_2022_2829_MOESM3_ESM.docx]

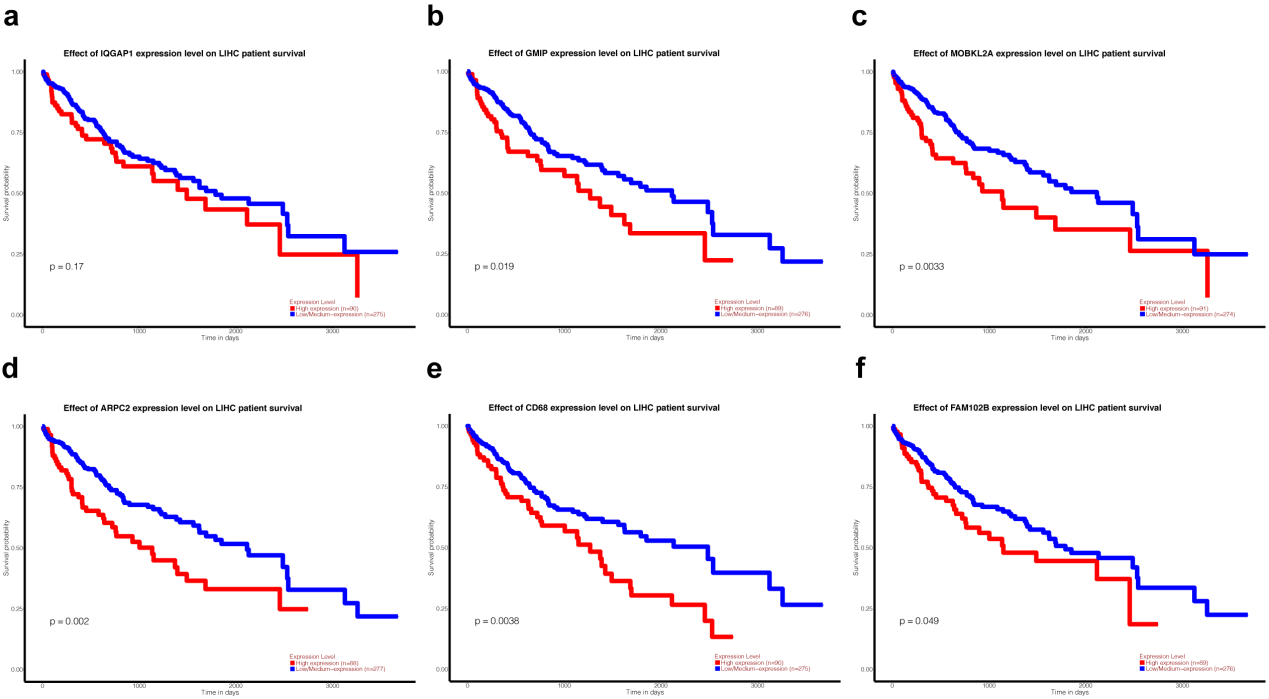

Supplement: Supplementary file 4 — Additional file 4: Figure S4. The prognostic value of CD97 co-expressed genes in HCC using the UALCAN database. a-f Correlations between OS and the mRNA levels of IQGAP1, GMIP, MOBKL2A, ARPC2, CD68, and FAM102B in HCC in the UALCAN database. [file 12957_2022_2829_MOESM4_ESM.docx]

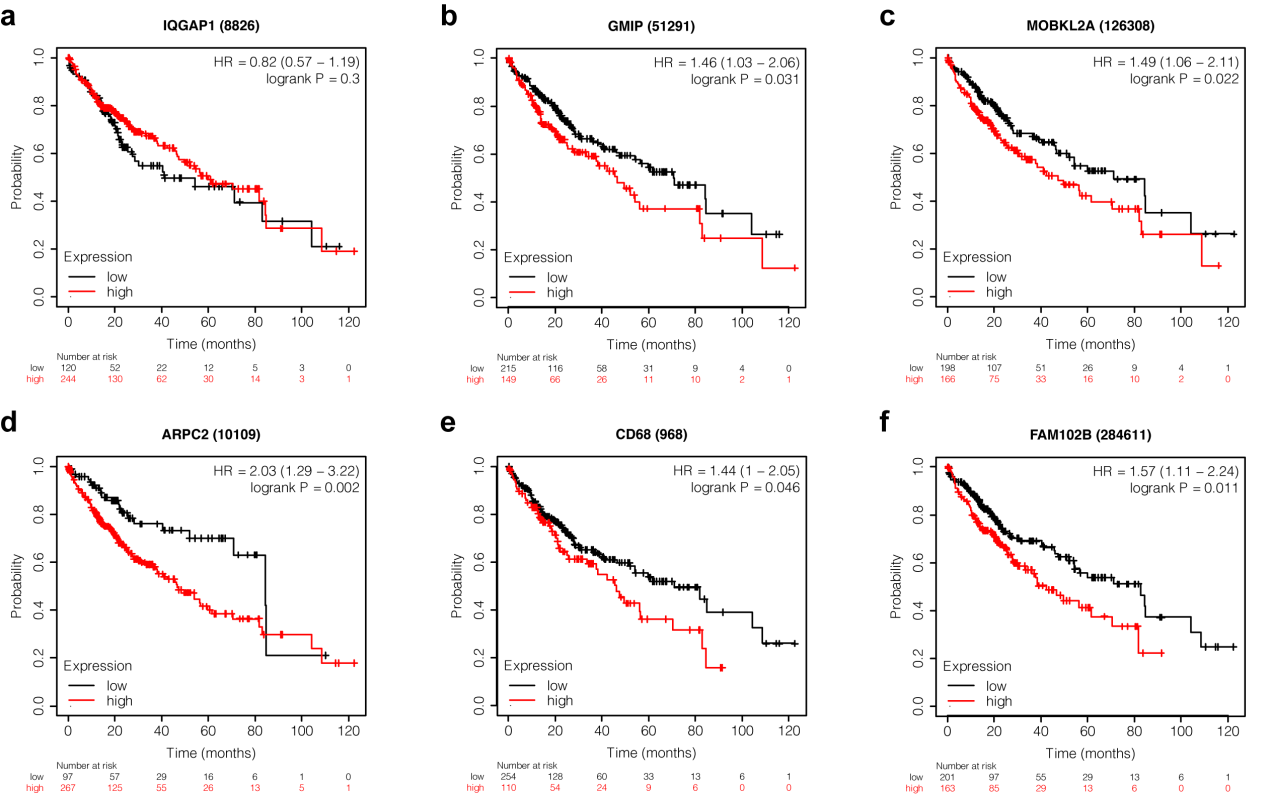

Supplement: Supplementary file 5 — Additional file 5: Figure S5. The prognostic value of CD97 co-expressed genes in HCC using the KM plotter database. a-f Correlations between OS and the mRNA levels of IQGAP1, GMIP, MOBKL2A, ARPC2, CD68, and FAM102B in HCC in the KM plotter database. [file 12957_2022_2829_MOESM5_ESM.docx]
