# Supplementary material for: CD97 serves as a novel biomarker of immune cell infiltration in hepatocellular carcinoma
Source: World J Surg Oncol. 2022 Dec 4;20:382. doi: 10.1186/s12957-022-02829-2 (PMC9721038; doi:10.1186/s12957-022-02829-2)
Supplement: Supplementary file 6 — Additional file 6: Table S1. GSEA pathways upregulated due to high expression of CD97. [file 12957_2022_2829_MOESM6_ESM.docx]

| **Table S1** GSEA pathways upregulated due to high expression of CD97. | | | | |
| --- | --- | --- | --- | --- |
| **Gene set name** | **SIZE** | **NES** | **NOM p-val** | **FDR q-val** |
| KEGG_LEISHMANIA_INFECTION | 69 | 1.90 | 0.000 | 0 |
| KEGG_GRAFT_VERSUS_HOST_DISEASE | 37 | 1.83 | 0.000 | 0 |
| KEGG_PRIMARY_IMMUNODEFICIENCY | 35 | 1.82 | 0.000 | 0 |
| KEGG_NOD_LIKE_RECEPTOR_SIGNALING_PATHWAY | 60 | 1.80 | 0.000 | 1.86E-04 |
| KEGG_INTESTINAL_IMMUNE_NETWORK_FOR_IGA_PRODUCTION | 46 | 1.79 | 0.000 | 1.49E-04 |
| KEGG_TYPE_I_DIABETES_MELLITUS | 41 | 1.78 | 0.000 | 2.50E-04 |
| KEGG_HEMATOPOIETIC_CELL_LINEAGE | 84 | 1.78 | 0.000 | 2.15E-04 |
| KEGG_ALLOGRAFT_REJECTION | 35 | 1.76 | 0.000 | 1.88E-04 |
| KEGG_CYTOKINE_CYTOKINE_RECEPTOR_INTERACTION | 258 | 1.73 | 0.000 | 4.26E-04 |
| KEGG_B_CELL_RECEPTOR_SIGNALING_PATHWAY | 74 | 1.72 | 0.000 | 4.60E-04 |
| KEGG_T_CELL_RECEPTOR_SIGNALING_PATHWAY | 106 | 1.71 | 0.000 | 6.26E-04 |
| KEGG_ASTHMA | 28 | 1.70 | 0.000 | 5.74E-04 |
| KEGG_CELL_ADHESION_MOLECULES_CAMS | 128 | 1.69 | 0.000 | 6.47E-04 |
| KEGG_CHEMOKINE_SIGNALING_PATHWAY | 185 | 1.69 | 0.000 | 6.56E-04 |
| KEGG_SYSTEMIC_LUPUS_ERYTHEMATOSUS | 54 | 1.69 | 0.000 | 7.14E-04 |
| KEGG_AUTOIMMUNE_THYROID_DISEASE | 50 | 1.66 | 0.000 | 1.24E-03 |
| KEGG_TOLL_LIKE_RECEPTOR_SIGNALING_PATHWAY | 100 | 1.64 | 0.000 | 1.70E-03 |
| KEGG_FC_GAMMA_R_MEDIATED_PHAGOCYTOSIS | 92 | 1.63 | 0.000 | 1.86E-03 |
| KEGG_DILATED_CARDIOMYOPATHY | 90 | 1.61 | 0.000 | 2.77E-03 |
| NES, normalized enrichment score; NOM, nominal; FDR, false discovery rate. Gene sets with NOM p-value < 0.05 and FDR q-value < 0.1 are considered as significant. | | | | |
